# Catheter-associated bacteremia by Mycobacterium senegalense in Korea

**DOI:** 10.1186/1471-2334-5-107

**Published:** 2005-11-25

**Authors:** Won Sup Oh, Kwan Soo Ko, Jae-Hoon Song, Mi Young Lee, Seong Yeol Ryu, Sangtaek Heo, Ki Tae Kwon, Jang-Ho Lee, Kyong Ran Peck, Nam Yong Lee

**Affiliations:** 1 Division of Infectious Diseases, Samsung Medical Center, Sungkyunkwan University School of Medicine, Seoul, Korea; 2 Department of Laboratory Medicine, Samsung Medical Center, Sungkyunkwan University School of Medicine, Seoul, Korea; 3 Asian-Pacific Research Foundation for Infectious Diseases (ARFID), Seoul, Korea

## Abstract

**Background:**

Rapidly growing mycobacteria is recognized as one of the causative agents of catheter-related infections, especially in immunocompromised hosts. To date, however, *Mycobacterium senegalense*, which was known as the principal pathogen of bovine farcy, has not been reported in human infection.

**Case presentation:**

We describe the first case of human infection by *M. senegalense*, which has caused catheter-related bloodstream infection in a cancer patient in Korea. The microorganism was identified by the 16S rRNA gene, *rpoB*, and 16S-23S rRNA gene internal transcribed spacer (ITS) sequence analyses.

**Conclusion:**

Our first report of catheter-associated bacteremia caused by *M. senegalense *suggests the zoonotic nature of this species and indicates the expansion of mycobacterial species relating to human infection. *M. senegalense *should be considered as one of the causes of human infections in the clinical practice.

## Background

*Mycobacterium senegalense *was originally described by Chamoiseau in 1973 as a subspecies of *Mycobacterium farcinogenes *[1]. However, it was later recognized as a distinct species closely related to *M. fortuitum *[2]. *M. senegalense *is known as the principal pathogen of bovine farcy, which is a chronic disease of skin and superficial lymphatics of cattle in East and Central Africa [3]. Unlike other rapidly growing mycobacteria, human infection by *M. senegalense *has not been reported to date. Here we report the first case of central venous catheter (CVC) infection caused by *M. senegalense*.

## Case presentation

A 49-year-old woman with non-Hodgkin's lymphoma was admitted to the hospital because of fever for several hours. The patient had been treated for lymphoma since 3 months ago. Five days before admission, the patient was treated with the third cycle of CHOP plus rituximab (R-CHOP). She had no history of travel or contact with animals including cows or their products. Physical examination revealed high fever of 39.8°C. The patient had a subclavian cuffed-CVC(Hickman catheter) on the right side with no evidence of the inflammation at the exit site. Laboratory data and chest radiograph were within normal limits. Three sets of blood samples for cultures were drawn through CVC lines (2 sets) and a peripheral vein (1 set), respectively. The patient was treated empirically with vancomycin (1 g every 12 h intravenously). On the second hospital day, all 3 sets of blood cultures grew gram-positive, acid-fast bacilli. The cultures from the CVC became positive more than 2 hours earlier than that from a peripheral vein. Non-pigmented and pinpoint-shaped colonies were observed on blood or chocolate agar plate after 3 days of incubation at 37°C. It did not grow well on McConkey agar plate. As it grows, the color of the colonies becomes pale-yellow. Vancomycin was replaced by imipenem/cilastatin (500 mg every 6 h intravenously) and amikacin (375 mg every 12 h intravenously). On the sixth hospital day, the CVC was removed because of persistent fever. After removal of CVC, the patient became afebrile and the repeated blood cultures became negative. In vitro susceptibility test was performed by broth microdilution test as described by the National Committee for Clinical Laboratory Standards (NCCLS) guidelines [4]. The result of in vitro susceptibility test was shown in Table 1. The isolates were susceptible to most antimicrobial agents tested except vancomycin. The patient was further treated with oral ciprofloxacin (500 mg every 12 hours) and doxycycline (100 mg every 12 hours) for 4 weeks. She had been doing well with no evidence of recurrence for the next 3 months.

**Table 1:** Antibiotic susceptibility testing using broth microdilution for strain SMC-7485.

Antibiotic agents	MIC (μg/mL)	Susceptibility ^a^
Amikacin	0.5	S
Cefoxitin	8	S
Ciprofloxacin	0.25	S
Clarithromycin	0.25	S
Doxycycline	0.12	S
Imipenem	4	S
Tobramycin	4	S
Amoxicillin-clavulanic acid	16/8	-
Moxifloxacin	0.12	S
Trimethoprim-sulfamethoxazole	4/76	-
Vancomycin	16	I

^a^S, susceptible; I, intermediate. MIC interpretative breakpoints of amoxicillin-clavulanic acid, moxifloxacin, trimethoprim-sulfamethoxazole, and vancomycin are not shown by NCCLS [4] for rapidly growing mycobacteria. Those of moxifloxacin and vancomycin are those recommended for aerobic organisms.

## Molecular identification

Conventional automated methods in the clinical microbiology laboratory such as VITEK 2 system (bioMérieux, Hazelwood, Mo.) failed to identify this isolate to a given species. Thus, this isolate ("SMC-7485"), was subjected to the 16S rRNA gene, *rpoB*, and 16S-23S rRNA gene internal transcribe spacer (ITS) sequence analyses for bacterial identification. Genomic DNA was extracted by using the G-Spin Genomic DNA Extraction Kit (iNtRON, Seoul, Korea). DNA amplification of 16S rRNA gene, *rpoB*, and ITS were performed by using primer sets 16S-F3 (5'-CAG GCC TAA CAC ATG CAA GT-3')/16S-R3 (5'-GGG CGG WGT GTA CAA GGC-3'), MF (5'-CGA CCA CTT CGG CAA CCG-3')/MR (5'-TCG ATC GGG CAC ATC CGG-3'), and ITS-F (TTG TAC ACA CCG CCC GTC A-3')/ITS-R (5'-TCT CGA TGC CCG GCA TCC ACC-3') [5-7], respectively. Template DNA (ca. 50 ng) and 20 pmol of each primer were added to a PCR mixture tube (AccuPower PCR PreMix; Bioneer, Daejeon, Korea) containing 1 unit of T*aq *DNA polymerase, each deoxynucleoside triphosphate at a concentration of 250 μM, 10 mM Tric-HCl (pH 8.3), 10 mM KCl, 1.5 mM MgCl_2_, and gel loading dye [8]. The reaction mixture was then subjected to 35 cycles for amplification. Each cycle consisted of 30 sec at 95°C for denaturation, 30 sec at 60°C, and 1 min at 72°C for extension, followed by final extension at 72°C for 5 min. Amplified PCR product was purified for sequencing using PCR purification kit (CoreOne, Seoul, Korea). The purified PCR product was sequenced directly using the same primers of PCR amplification at both directions. Sequence editing and analyses were performed with the EditSeq and MegAlign programs in DNASTAR (Windows version 3.12e; Madison, Wis.). Determined sequence was compared with public database, GenBank, with the BLASTn program , and sequences showing high similarity were retrieved for further analysis.

16S rRNA gene sequence (1,393 bp) of the strain SMC-7485 showed 100% similarities with those of *Mycobacterium senegalense *ATCC 35796 [GenBank: AF480596] and *Mycobacterium farcinogenes *ATCC 35753 [GenBank: AF055333]. It also showed very high similarities (99.5% – 99.7%) with those of *Mycobacterium porcinum, Mycobacterium housetonense, Mycobacterium neworleansense, Mycobacterium boenickei, Mycobacterium septicum*, and *Mycobacterium fortuitum*. 16S rRNA gene sequence analysis suggested that the strain SMC-7485 belong to *M. senegalense *or *M. farcinogenes *of *M. fortuitum *group. However, more decisive identification could not be available due to no divergence of 16S rRNA gene sequence between two species [9].

**Figure 1:**
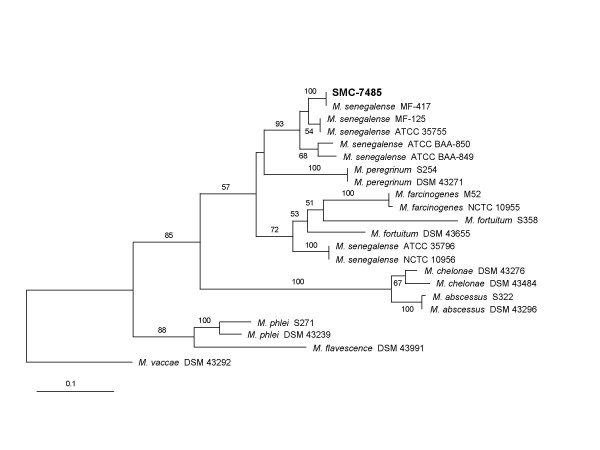
Phylogenetic relationships of SMC-7485 and other *Mycobacterium *species based on ITS sequences, which were retrieved from GenBank database. This tree was generated by the neighbor-joining method. *Mycobacterium vaccae *DSM 43292 was used as an outlier. Numbers at branching nodes are percentages of 1,000 bootstrap replications. Only values greater than 50% are shown. In this tree, *M. senegalense *strains are separated into two subgroups.

To clarify identification of the strain SMC-7485, we analyzed *rpoB *and ITS sequences. *rpoB *gene sequence (301 bp) of SMC-7485 showed that it was the closest to *M. senegalense *ATCC 35796 [GenBank: AF057483], i.e. 99.7% similarity. The species showing the next highest similarities were *M. porcinum, Mycobacterium wolinskyi, Mycobacterium goodii*, and *M. septicum *(96.9% – 99.0%). On the other hand, *rpoB *gene sequence of *M. farcinogenes *DSM 43637 [GenBank: AY544910] was diverged from that of SMC-7485 (94.7% similarity). ITS sequence (327 bp) analysis also suggested that the strain SMC-7485 belonged to *M. senegalense*. The strain SMC-7485 showed an identical ITS sequence with *S. senegalense *MF-417 [GenBank: AY684051]. In addition, ITS sequence of SMC-7485 showed the similarities of more than 90% with those of several *M. senegalense *strains deposited in GenBank database. Although ITS sequences of some *M. senegalense *strains such as ATCC 35796 and NCTC 10956 showed low similarities (80.9%) with that of SMC-7485, they were clearly differentiated from *M. farcinogenes *and other *Mycobacterium *species (Fig. 1). Based on 16S rRNA gene, *rpoB *gene, and ITS sequences, we could identify SMC-7485 as *M. senegalense*.

The nucleotide sequences of the 16S rRNA gene, *rpoB*, and ITS of the strain SMC-7485 have been deposited in the GenBank database under accession numbers DQ145802 to DQ145804.

## Discussion

Rapidly growing mycobacteria such as the *M. fortuitum *group, the *M. chelonae/abscessus *group, and the *M. smegmatis *group are capable of thriving in even the most hostile environments [10]. Due to their ubiquitous capability, human infections by the rapidly growing mycobacteria have been identified with increasing frequency worldwide. Especially, rapidly growing mycobacteria are being recognized as one of the significant pathogens of catheter-related infections in immunocompromised hosts. Among rapidly growing mycobacteria, the *M. fortuitum *group is the most common mycobacterial pathogen for this clinical condition [10,11].

The *M. fortuitum *group included *M. fortuitum, M. peregrinum*, *M. mucogenicum, M. senegalense, M. mageritense*, and several newly described species such as *M. septicum, M. houstonense*, *M. boenickei, M. neworleansense*, and *M. brisbanense *[12]. Of these, *M. senegalense *was originally described by Chamoiseau in 1973 as a subspecies of *M. farcinogenes*. Although *M. farcinogenes *and *M. senegalense *have identical 16S rRNA gene sequences, *M. senegalense *could be identified as a different species based on differences in growth rate, chemical activity and DNA homology [1,6,13,14]. While most species of *M. fortuitum *group have been reported to be responsible for various human diseases, human infection by *M. senegalense *has not been described to date [3,10,12]. Instead, it causes the chronic infectious disease of zebu cattle known as bovine farcy, endemic to East and Central Africa [3,9]. Moreover, *M. senegalense*, which was originally found in Africa, has never been described elsewhere [3,10].

In this report, we have first documented the CVC infection caused by *M. senegalense*. Because conventional automated methods failed to identify it at the species level, we tried to sequence 16S rRNA gene, *rpoB *gene, and ITS region. By 16S rRNA gene, *rpoB *gene, and ITS sequence analyses, we concluded that an agent of CVC infection in our patient was *M. senegalense*. *rpoB *gene and ITS sequences could differentiate *M. senegalense *from *M. farcinogenes *clearly as in previous reports [6,14]. Moreover, ITS sequence analysis indicated that *M. senegalense *might consist of at least two heterogeneous groups (Fig. 1). There is possibility that *M. senegalense *isolate related to human infection has been misidentified as different species because it is difficult to identify nontuberculous mycobacteria (NTM) at the species level [9,16]. However, the strain SMC-7485 is the first described *M. senegalense *isolate, which is associated with human infection and is found outside Africa, to our knowledge.

In most cases with mycobacterial infection of CVC, the line should be removed for successful control of infection [17]. In this study, the patient failed to respond to an initial regimen of imipenem and amikacin, to which the isolate was susceptible. Persistent infection was controlled after removal of catheter, which emphasized the importance of catheter removal. Because of differences in susceptibilities among species and even within species, rapid identification and subsequent susceptibility testing are essential for selection of appropriate antibiotic agent(s) against rapidly growing mycobacteria [18]. In this study, the strain SMC-7485 was susceptible to amikacin, cefoxitin, ciprofloxacin, clarithromycin, doxycycline, and imipenem. Because of the high frequency of relapse and resistance, combination therapy with multiple antibiotics is usually recommended for serious infections by rapidly growing mycobacteria. However, the optimal antibiotic regimen has yet to be defined for catheter-related infection by these mycobacteria. In our experience, oral antibiotic therapy subsequent to a short course of intravenous antibiotics seemed to be effective and safe. Although it was not possible to determine the optimal duration of antibiotic therapy in our case, this episode was successfully treated with short-term (approximately 5 weeks) antibiotic therapy. Further studies will be required to confirm these findings.

## Conclusion

In this paper, we firstly reported catheter-associated bacteremia by *M. senegalense*. This case suggested that *M. senegalense *can cause human infections. This pathogen should be included in the list of nontuberculous mycobacteria causing human infections.

## List of abbreviations

CVC – central venous catheter

ITS – internal transcribed spacer

MIC – minimum inhibitory concentration

NCCLS – National Committee for Clinical Laboratory Standards

NTM – nontuberculous mycobacteria

## Competing interests

The author(s) declare that they have no competing interests.

## Authors' contributions

WSO, SYR, KTK, and STH followed up the patient and obtained consent from the patient for this case report. WSO, KRP, and NYL provided clinical details. KSK performed the molecular identification and phylogenetic analysis, and KSK, MYL, and JHL executed antimicrobial susceptibility testing. WSO, KSK, and JHS drafted the manuscript. All authors read and approved the final manuscript.

## Pre-publication history

The pre-publication history for this paper can be accessed here:


